# Pediatric periorificial dermatitis in an Asian population: A comparative study of oral metronidazole and oral macrolides

**DOI:** 10.1016/j.jdin.2024.05.005

**Published:** 2024-05-31

**Authors:** Jeewoo Kang, Youngjoon Ahn, Joong Heon Suh, Seon-Pil Jin

**Affiliations:** a Department of Dermatology, Seoul National University College of Medicine, Seoul, Republic of Korea; b Department of Dermatology, Seoul National University Hospital, Seoul, Republic of Korea; c Department of Medicine, Seoul National University College of Medicine, Seoul, Republic of Korea; d Institute of Human-Environment Interface Biology, Medical Research Center, Seoul National University, Seoul, Republic of Korea

**Keywords:** oral macrolides, oral metronidazole, pediatric periorificial dermatitis, periorificial dermatitis, PPD, topical calcineurin inhibitors, topical metronidazole

*To the Editor:* A diverse array of therapeutic agents is available for pediatric periorificial dermatitis (PPD).^1^ However, PPD management is mostly supported by nonrandomized studies predominantly involving the Caucasian population, with limited data on the comparison of efficacy between the various oral antibiotics. Therefore, we aimed to address this gap by delineating PPD characteristics in an Asian population and evaluating treatment outcomes of systemic antibiotics, specifically comparing oral metronidazole (OMZ) to oral macrolides.

This is a retrospective cohort study of pediatric patients aged 0 to 18 years diagnosed with periorificial dermatitis using the diagnostic code L71.0 (International Classification of Diseases, 10th revision) at a single pediatric dermatology clinic from January 1, 2014, to June 30, 2023. Severity was evaluated using the pediatric periorificial dermatitis severity index.^2^ Severity grades were determined by the total pediatric periorificial dermatitis severity index score, with classifications as follows: 0 as clear, 1 to 3 as mild, 4 to 5 as moderate, and 6 to 7 as severe. Time to improvement of the severity grade was calculated as the duration of time from treatment initiation to the first recorded date of at least a one-level reduction in severity grade.

Overall, 159 patients were included, with a slightly higher prevalence in males (55%) and a mean (standard deviation) onset age of 6.7 (3.0) years (Table I). Topical steroid use was the most common patient-reported triggering factor (53.5%), followed by systemic steroids (12.6%).

**Table I:** Demographics and clinical characteristics of pediatric patients with pediatric periorificial dermatitis (*n* = 159)

Demographics and clinical characteristics	
Number of patients, *n*	159
Sex, *n* (%)	
Male	88 (55.3)
Female	71 (44.7)
Onset age, mean (SD)	6.7 (3.0)
Location of lesions, *n* (%)∗	
Perioral	126 (95.4)
Periorbital	76 (57.6)
Perinasal	109 (82.6)
Number of periorificial areas involved, *n* (%)∗	
1	16 (12.1)
2	53 (40.2)
3	63 (47.7)
Baseline PPODSI, mean (SD)∗	3.8 (1.4)
Baseline severity, *n* (%)∗	
Mild	54 (40.9)
Moderate	53 (40.2)
Severe	25 (18.9)
Patient-reported triggering factors, *n* (%)	
Topical steroids	85 (53.5)
Systemic steroids	20 (12.6)
Others†	2 (1.3)
Comorbidities, *n* (%)	
Atopic dermatitis	23 (14.5)
Allergic rhinitis	5 (3.1)
Asthma	1 (0.6)
Malignancy (ALL)	1 (0.6)
Treatment received, *n* (%)	
Topical calcineurin inhibitors	132 (83.0)
Topical metronidazole	108 (67.9)
Oral antibiotics	88 (55.3)

*ALL*, Acute lymphoblastic leukemia; *n*, number of patients; *PPODSI*, pediatric periorificial dermatitis severity index; *SD*, standard deviation.

∗ Among those with clinical photographs available at initial visit (*n* = 132).

† Includes 1 patient reporting nasal steroid spray use and 1 patient reporting COVID-19 as the potential triggering factor.

Of the total patients, 56 patients who had standardized clinical photographs at both treatment initiation and completion were assessed regarding their treatment outcome. Their course of treatment involved either OMZ at 15 to 35 mg/kg/day (*n* = 34) or oral macrolides, including roxithromycin at 5 to 8 mg/kg/day (*n* = 11), clarithromycin at 7.5 mg/kg twice a day (*n* = 7), and azithromycin at 5 to 10 mg/kg 3 times a week (*n* = 4) (Table II). All the assessed patients underwent routine 2- to 4-week interval follow-ups while on oral antibiotic therapy and were concomitantly treated with topical calcineurin inhibitors (TCIs) and/or topical metronidazole (TMZ). Most patients maintained topical therapies after oral antibiotic cessation to prevent recurrence or aggravation until treatment completion. Nearly all patients achieved at least a partial response (macrolides, 90.9%; OMZ, 97.1%). Treatment outcomes were comparable between the 2 groups, but the macrolides group showed a shorter time to severity improvement (macrolides, 21.4 days vs OMZ, 41.8 days; *P* < .001) and a shorter duration of oral antibiotics use (macrolides, 49.6 days vs OMZ, 87.3 days; *P* < .001). Dose reduction was necessary in one patient per treatment group due to recurrent diarrhea, with no further recurrences after dose adjustments.

**Table II:** Comparison of treatment outcomes between patients treated with oral metronidazole and patients treated with oral macrolides (*n* = 56)

Treatment outcomes	OMZ + TCI/TMZ (*n* = 34)		Macrolides + TCI/TMZ (*n* = 22)		*P* value∗
	Baseline	End of treatment	Baseline	End of treatment	
PPODSI, mean (SD)	4.6 (1.3)	1.1 (1.3)†	4.5 (1.5)	1.1 (1.4)†	
Post-treatment PPODSI reduction, %, mean (SD)	79.5 (25.8)		78.3 (29.2)		.87
Severity, *n* (%)					
Clear	0 (0.0)	19 (55.9)	0 (0.0)	12 (54.5)	
Mild	4 (11.8)	13 (38.2)	5 (22.7)	9 (40.9)	
Moderate	18 (52.9)	3 (5.9)	8 (36.4)	1 (4.5)	
Severe	12 (35.3)	0 (0.0)	9 (40.9)	0 (0.0)	
Treatment response, *n* (%)					
Complete response‡	19 (55.9)		12 (54.5)		.67
Partial response§	14 (41.2)		8 (36.4)		
No response‖	1 (2.9)		2 (9.1)		
Recurrence¶	4 (11.8)		2 (9.1)		1
Duration of treatment, days, mean (SD)					
Oral antibiotics	87.3 (41.0)		49.6 (28.2)		**<.001**
Topical treatments	138.9 (74.2)		110.1 (77.2)		.17
Time from treatment initiation to severity improvement, days, mean (SD)	41.8 (22.6)#		21.4 (9.3)∗∗		**<.001**
Concomitant topical treatments, *n* (%)					
TCI	34 (100.0)		19 (86.4)		
TMZ	31 (91.1)		11 (50.0)		
Adverse events, *n* (%)					
Diarrhea	1 (2.9)		2 (9.1)		

*n*, Number of patients; *OMZ*, oral metronidazole; *PPODSI*, pediatric periorificial dermatitis score index; *SD*, standard deviation; *TCI*, topical calcineurin inhibitors; *TMZ*, topical metronidazole.

∗ Bold *P* values are statistically significant (*P* < .05). *P* values were calculated using the independent *t*-test and Fisher's exact test.

† Statistically significant differences were found between PPODSI before and after treatment using the paired *t*-test (OMZ, *P* < .001; macrolides, *P* < .001).

‡ Reduction of PPODSI to 0 (clear).

§ At least a one-level reduction in severity grade without achieving a clear state.

‖ No changes in severity grade or an increase in PPODSI.

¶ Recurrence from patients who achieved a complete response.

# Excluded 1 patient who showed no response to OMZ + TCI/TMZ treatment (*n* = 33).

∗∗ Excluded 2 patients who showed no response to oral macrolides + TCI/TMZ treatment (*n* = 20).

Limitations of the study include its retrospective design, variations in concomitant TCI/TMZ use, and the diverse selection of macrolides. Nevertheless, it comprises the largest pediatric PPD cohort within an Asian population to date, which is noteworthy since previous research has predominantly focused on the Caucasian population.^3^, ^4^, ^5^

While acknowledging the need for larger, controlled studies, these findings suggest that both OMZ and macrolides may be effective options for PPD. Owing to its more rapid treatment response and shorter treatment duration, oral macrolides with concomitant TCI/TMZ may be initially considered over OMZ with concomitant TCI/TMZ. This is particularly notable in situations where patients and caregivers show hesitancy toward prolonged use of systemic antibiotics.

## Conflicts of interest

None disclosed.

## References

[1] Gray N.A., Tod B., Rohwer A., Fincham L., Visser W.I., McCaul M. (2022). Pharmacological interventions for periorificial (perioral) dermatitis in children and adults: a systematic review. J Eur Acad Dermatol Venereol.

[2] Lee H., Kim K.H. (2021). Treatment of pediatric periorificial dermatitis with topical calcineurin inhibitor and topical/oral metronidazole. J Dermatol.

[3] Nguyen V., Eichenfield L.F. (2006). Periorificial dermatitis in children and adolescents. J Am Acad Dermatol.

[4] Goel N.S., Burkhart C.N., Morrell D.S. (2015). Pediatric periorificial dermatitis: clinical course and treatment outcomes in 222 patients. Pediatr Dermatol.

[5] Ollech A., Yousif R., Kruse L. (2020). Topical calcineurin inhibitors for pediatric periorificial dermatitis. J Am Acad Dermatol.

